# Mitochondrial Role on Cellular Apoptosis, Autophagy, and Senescence during Osteoarthritis Pathogenesis

**DOI:** 10.3390/cells13110976

**Published:** 2024-06-04

**Authors:** Andrea Dalmao-Fernández, Tamara Hermida-Gómez, Uxia Nogueira-Recalde, Ignacio Rego-Pérez, Francisco J. Blanco-Garcia, Mercedes Fernández-Moreno

**Affiliations:** 1 Grupo de Investigación en Reumatología (GIR), Instituto de Investigación Biomédica de A Coruña (INIBIC), Complexo Hospitalario Universitario de A Coruña (CHUAC), Sergas, Universidade de A Coruña (UDC), 15071 A Coruña, Spain; a.d.fernandez@farmasi.uio.no (A.D.-F.); tamara.hermida.gomez@sergas.es (T.H.-G.); uxia.nogueira.recalde@sergas.es (U.N.-R.); ignacio.rego.perez@sergas.es (I.R.-P.); 2 Section for Pharmacology and Pharmaceutical Biosciences, Department of Pharmacy, University of Oslo, 0316 Oslo, Norway; 3 Grupo de Investigación en Reumatología y Salud (GIR-S), Centro Interdisciplinar de Química y Biología (CICA), Universidade de A Coruña (UDC), Campus de Elviña, 15071 A Coruña, Spain; 4 Centro de Investigación Biomédica en Red, Bioingenieria, Biomatereiales y Nanomedicina (CIBER-BBN), 28029 Madrid, Spain; 5 Grupo de Investigación en Reumatología y Salud (GIR-S), Departamento de Fisioterapia, Medicina y Ciencias Biomédicas, Facultad de Fisioterapia, Centro Interdisciplinar de Química y Biología (CICA), INIBIC-Sergas, Universidade de A Coruña (UDC), Campus de Oza, 15008 A Coruña, Spain

**Keywords:** apoptosis, autophagy, senescence, transmitochondrial cybrids, mitochondria, osteoarthritis

## Abstract

Authors have demonstrated that apoptosis activation is a pathway related to cartilage degradation characteristics of the OA process. Autophagy is an adaptive response to protect cells from various environmental changes, and defects in autophagy are linked to cell death. In this sense, decreased autophagy of chondrocytes has been observed in OA articular cartilage. The aim of this work was to study the role of OA mitochondria in apoptosis, autophagy, and senescence, using OA and Normal (N) transmitochondrial cybrids. Results: OA cybrids incubated with menadione showed a higher percentage of late apoptosis and necrosis than N cybrids. Stimulation of cybrids with staurosporine and IL-1β showed that OA cybrids were more susceptible to undergoing apoptosis than N cybrids. An analysis of the antioxidant response using menadione on gene expression revealed a lower expression of nuclear factor erythroid 2-like 2 and superoxide dismutase 2 in OA than N cybrids. Activation of microtubule-associated protein 1A/1B-light chain 3 was reduced in OA compared to N cybrids. However, the percentage of senescent cells was higher in OA than N cybrids. Conclusion: This work suggests that mitochondria from OA patients could be involved in the apoptosis, autophagy, and senescence of chondrocytes described in OA cartilage.

## 1. Introduction

Recent insights indicate that the conventional understanding of osteoarthritis (OA) solely as a consequence of aging or mechanical stress on the joints is no longer adequate to comprehend its underlying mechanisms [1,2]. The pathological transformations observed across all joint tissues prompt us to view OA not merely as a localized joint disorder, but rather as a systemic disease affecting the entire joint as an organ [3,4]. While initial joint injury inflicts direct harm to joint tissues, a significant portion of subsequent damage stems from cellular responses to the injury [5]. By reconceptualizing OA and recognizing the joint as an organ, it becomes apparent that this condition entails systemic implications with a backdrop of mild, chronic inflammation [6].

Such a framework holds promise in uncovering innovative strategies for maintaining cellular equilibrium during the aging process. Recent investigations have extensively reviewed the role of oxidative stress and aberrant redox signaling in the pathophysiology of OA, revealing heightened levels of oxidative damage to proteins, lipids, and DNA in cartilage and other joint tissues [7,8,9,10].

Mitochondrial DNA (mtDNA) haplogroups correlate with OA in several cohorts from different countries [11]. Several articles have described the association between mtDNA variants and the OA [12,13,14]. Another study described, for the first time, the association between the mtDNA variants and the methylation status in articular cartilage by acting on key mechanisms involved inOA, such as apoptosis and metabolic and developmental processes [15].

Several cellular events participate during the cartilage degradation and OA pathogenesis such as apoptosis, autophagy, mitogenesis, and senescence. Several authors demonstrated that apoptosis activation plays an important role in OA pathogenesis [16,17]. Autophagy involves the engulfment of cytoplasmic contents into double membrane-bound vesicles called autophagosomes, which fuse with lysosomes, degrading their contents, which are subsequently then released into the cytoplasm [18]. Autophagy acts as an adaptive response to protect chondrocytes from various environmental changes, while, with gradual cartilage degradation, decreased autophagy is linked with cell death [19]. Defects in this process have been observed in OA articular cartilage [20,21]. Autophagy is essential to preserve the integrity and function of articular cartilage [22].

Evidence indicates that mitochondria are involved in the pathogenesis of OA [23]. The maintenance of mitochondria homeostasis and function is important for the correct function of tissues. The elimination of damaged mitochondria occurs by mitophagy [24]. This is a selective process that removes damaged or dysfunctional mitochondria through autophagic machinery, and functions to maintain mitochondrial quality and homeostasis [25].

Senescence is characterized by proliferation arrest, an increase in cell size and mitochondrial mass together with mitochondrial dysfunction, and the increased secretion of pro-inflammatory and pro-oxidant signals [26,27,28]. The concept of “chondrosenescence” was defined some years ago and is intimately linked with the OA process since this disturbed the balance between autophagy and inflammasomes, contributing to the age-related degradation of articular cartilage and other joint tissues [29,30].

Mitochondrial fission and fusion orchestrate the quantity, distribution, and morphology of these cellular organelles, thereby exerting significant influence on various mitochondrial functions including energy generation, metabolic processes, intracellular signaling, and apoptosis [31,32,33]. The fission process, essential for generating new mitochondria, is governed by mitochondrial fission protein 1 (*Fis 1*) [34]. Conversely, mitochondrial fusion is regulated by GTPase enzymes, specifically mitofusins 1 and 2 (*Mfn1* and *Mfn2*), which oversee fusion of the outer mitochondrial membrane. Maintaining a delicate balance between fission and fusion mechanisms is pivotal for the upkeep of the mitochondrial structure and functionality in healthy cells. Crosstalk between apoptosis, autophagy, mitophagy, and mitochondrial fission and fusion seems to be critical for cells upon the induction of cell death. All data have shown that the different modes of cell death are not exclusive, but, rather, modulate each other [35].

Transmitochondrial cybrids are generated by fusing a cell without mitochondrial DNA (mtDNA) (Rho-0 cell) with an enucleated cell that harbors the mitochondria DNA of interest (platelets or enucleated fibroblasts). This cellular model is interesting because it allows for the study of the real role of mitochondria with the same nuclear DNA background [36,37]. Cybrids have been successfully used to explore the contribution of mitochondrial dysfunction and/or mtDNA gene mutations to the pathogenesis of diseases. These diseases include Parkinson’s, Alzheimer’s, mitochondrial cardiomyopathy, mitochondrial encephalopathy lactic acidosis stroke-like episodes (MELAS), and Myoclonic Epilepsy with Ragged-Red Fibers (MERF). To our knowledge, we are the first researchers to use cybrids as an in vitro model to describe the functional relationship between mitochondria alteration and OA [14,36,37].

The aim of this work was to study the role of OA mitochondria in apoptosis, autophagy, and senescence using transmitochondrial cybrids.

## 2. Materials and Methods

### 2.1. Participants

Platelet samples from both healthy individuals (N) and those with osteoarthritis (OA) were sourced from the Sample Collection for Rheumatic Disease Research, established by Dr. Blanco and recorded in the National Biobank Registry under Collection Code C.0000424. All participants provided written informed consent, and the study received approval from the local Ethics Committee of the Galician Health Administration. The procedures adhered to the principles outlined in the Declaration of Helsinki of 1975, as revised in 2000.

### 2.2. Transmitochondrial Cybrid Preparation

Cybrids generated from the 143B.TK-Rho-0 cell line were created following the methods detailed in prior studies [36,37]. Transmitochondrial cybrids were cultured in Dulbecco’s Modified Eagle Medium (DMEM, Gibco, Grand Island, NY, USA) with 10% fetal bovine serum (FBS), penicillin (100 U/mL), and streptomycin (100 μg/mL; Gibco). For experiments requiring metabolic stress conditions, cells were grown in low glucose DMEM (1 g/L). The cells were incubated at 37 °C in a humidified atmosphere with 5% CO_2_. For each experiment, cells from two healthy donors (2 N) and two osteoarthritic donors (2 OA), with two clones from each (4 N and 4 OA), were analyzed. Experiments were conducted using cells at passages 27–34.

### 2.3. Detection of Apoptotic Cells

The susceptibility of cells to apoptosis was analyzed. Cells were incubated in the presence of 2 µM staurosporine, a protein kinase inhibitor, for 2 h, 10 ng/mL interleukin 1β (IL-1β) for 48 h, or 50 µM menadione, a phosphatase inhibitor and an inhibitor of mitochondrial DNA polymerase γ, for 2 h (Sigma–Aldrich, Merck KGaA, Darmstadt, Germany). Cells were resuspended in a 1× annexin-binding buffer prior to adding 5 μL of annexin V–fluorescein isothiocyanate (FITC) and 5 μL of propidium iodide (PI; ImmunoStep, Salamanca, Spain).

After 15 min of incubation, 400 μL of the 1× annexin-binding buffer was added before analysis with a FACsCalibur flow cytometer (Becton Dickinson, Franklin Lakes, NJ, USA). For each assay, 1 × 10^4^ cells were measured. The data were processed using CellQuest software (version 7.5.3, Becton Dickinson). Apoptosis was assessed by counting cells that were stained with both annexin V–FITC and PI. The results were expressed as the percentage of cells positive for each stain.

### 2.4. Analysis of Anion Superoxide Production

To evaluate the role of menadione in oxidative stress, the mitochondrial anion superoxide (O_2_^−^) was measured. Cells were treated with 50 µM menadione for 2 h (Sigma–Aldrich), and 5 µM MitoSOX™ Red (Invitrogen, Carlsbad, CA, USA) was then added. Cells (1 × 10^4^) per assay were measured by FACsCalibur flow cytometry (Becton Dickinson), and data were analyzed using CellQuest software (Becton Dickinson). Results were expressed as the mean of the median of fluorescence (arbitrary units, AU) from three independent experiments.

### 2.5. Autophagy Determination

Autophagy was assessed through the analysis of LC3 and phospho-ribosomal protein S6 (p-rpS6) using western blotting (WB). To serve as a positive control for LC3, cells were incubated with 30 µM chloroquine (Sigma–Aldrich) for 16 h. For p-rpS6, cells were treated with 10 µM rapamycin (Calbiochem, Germany) for 16 h. The following antibodies were employed: LC3 (1:1000; Cell Signaling Technology, Beverly, MA, USA; #3868), p-rpS6 (1:2000; Cell Signaling Technology; #4858), and α-tubulin (1:5000; Sigma–Aldrich; #T9026). Band intensity was analyzed using Amersham Imager 600 software, and protein quantification on WB was performed using Image J v1.54g software Java8 (https://imagej.net/ij/, accessed on 23 May 2024).

### 2.6. Senescence Determination

The percentage of senescent cells was evaluated by the quantification of ẞ-galactosidase activity and *CDKN1A* gene expression. β-galactosidase activity was detected using fluorescein di-β-D-galactopyranoside (FDG-10 µM; Thermo Fisher, Waltham, MA, USA) by flow cytometry. Cell cultures were pretreated with 2 µM etoposide (Sigma–Aldrich) for 48 h to induce DNA damage as a genotoxic stress leading to cellular senescence, and 10 nM bafilomycin A (Sigma–Aldrich) was then added for 1 h to modulate intracellular pH. At the end of incubation, cultures were washed with PBS, resuspended by trypsinization, and analyzed immediately using a FACScalibour flow cytometer (Becton Dickinson) as described before.

### 2.7. Quantitative Real-Time PCR

Total RNA was extracted with TRIzol (Life Technologies, Carlsbad, CA, USA), and 0.5 µg was reverse transcribed into cDNA using the NZY First-Strand cDNA Synthesis Kit (NZY Tech, Lisboa, Portugal). Quantitative real-time PCR (qRT-PCR) was performed using a LightCycler 480-II Instrument (Roche, Mannheim, Germany) along with the LightCycler 480 Probe Master (Roche). The results were analyzed using Qbase+ version 2.5 software (Biogazelle, Gent, Belgium). Gene expression was calculated relative to the reference gene glyceraldehyde-3-phosphate dehydrogenase (*GAPDH*). Sequence primers and universal probe library (UPL) probes are described in Table 1.

### 2.8. Statistical Analysis

Statistical analyses were performed using GraphPad Prism version 8.0.1 for Windows (GraphPad Software, San Diego, CA, USA). Data are presented as the mean ± standard error of the mean (SEM) from independent experiments with a minimum of three observations unless stated otherwise. An unpaired Mann–Whitney *U* test was used to evaluate differences between groups. Differences with *p* values ≤ 0.05 were considered to be statistically significant.

## 3. Results

### 3.1. Apoptosis Analysis

**Figure 1:**
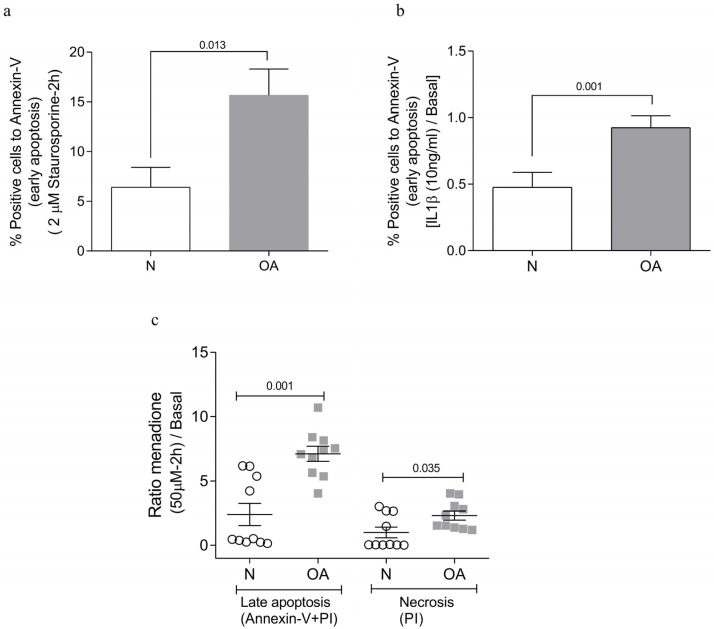
Analysis of apoptotic cells. Cybrids cells were treated with different stimuli. (**a**) Staurosporine (2 µM). (**b**) Inflammatory environment (IL-1β, 10 ng/mL). (**c**) Mitochondrial stress in the presence of menadione (50 µM). All data were obtained from three independent experiments performed with two replicates and two clones of each donor. Data are presented as mean ± SEM and analyzed by unpaired Mann–Whitney *U* test. PI: propidium iodide; N: healthy cybrids; OA: osteoarthritis cybrids.

The analysis of apoptotic levels showed that all cybrids had the same percentage of positive cells for annexin-V not exceeding 5.51% under basal conditions. However, when the cells were treated with staurosporine, a classical apoptotic stimulus, OA cybrids showed a statistically significant increase in positive cells for annexin-V in comparison to N cybrids (*p* = 0.013) (Figure 1a). To evaluate the cybrids’ response in an inflammatory environment, cells were treated with IL-1β. It was found that OA cybrids were more susceptible to undergoing apoptosis than N cybrids (*p* = 0.001) (Figure 1b).

To test the survival capacity of cybrids under mitochondrial stress, 50 µM menadione was used. Higher concentrations of menadione induced toxic oxidant stress associated with tissue injury, mitochondrial DNA damage, and cell death [38]. When the OA cybrids were incubated with menadione, the percentage in late apoptosis (*p* = 0.001) and necrosis (*p* = 0.03) was higher in OA cybrids than in N cybrids (Figure 1c). Early apoptosis was similar in both OA and N cybrids.

**Figure 2:**
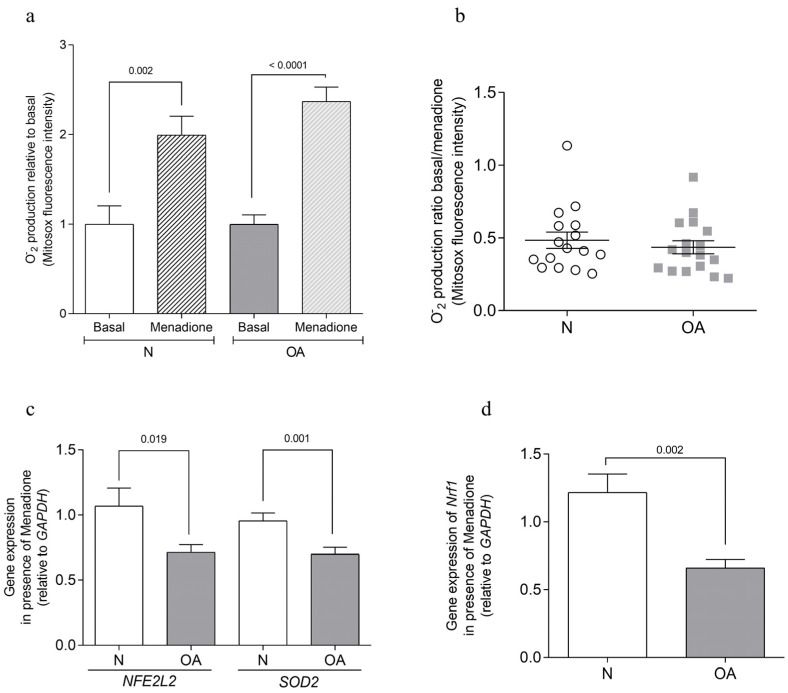
Role of menadione in oxidative stress. (**a**) Mitochondrial anion superoxide (O_2_^−^) production under basal and menadione conditions was evaluated by the fluorescence intensity of MitoSox^®^. (**b**) Data expressed as the ratio of basal/menadione O_2_^−^ between N and OA. (**c**) Relative mRNA expression levels in the presence of menadione (50 µM), nuclear factor erythroid 2-like 2 (*NFE2L2*), and superoxide dismutase-2 (*SOD2*) were detected by quantitative real-time (RT)–PCR. (**d**) Relative mRNA expression level in the presence of menadione of nuclear respiratory factor 1 (*Nrf1*) was detected by RT–PCR. All data were obtained from four independent experiments performed in duplicate. Data are presented as mean ± SEM and analyzed by unpaired Mann–Whitney *U* test. N: healthy cybrids; OA: osteoarthritis cybrids.

The effect of 50 µM menadione on oxidative stress was assessed by measuring O_2_^−^ production after 2 h of cell incubation and evaluating the antioxidant response after 48 h of exposure. Menadione treatment resulted in increased O_2_^−^ levels in both healthy (N) (*p* = 0.002) and osteoarthritic (OA) (*p* ≤ 0.0001) cybrids (Figure 2a). However, no significant differences were observed in the basal/menadione ratio between N and OA groups (Figure 2b). The antioxidant response, assessed by the gene expression of nuclear factor erythroid 2-like 2 (*NFE2L2*) and superoxide dismutase 2 (*SOD2*), showed a lower expression in OA cybrids compared to N cybrids (*p* = 0.019 and *p* = 0.001, respectively) when cultured with menadione (Figure 2c). Additionally, the analysis of mitochondrial biogenesis, through the expression of nuclear respiratory factor 1 (*Nrf1*), indicated a reduced level in OA cybrids compared to N cybrids (*p* = 0.002) in the presence of menadione (Figure 2d).

### 3.2. Autophagy Analysis

**Figure 3:**
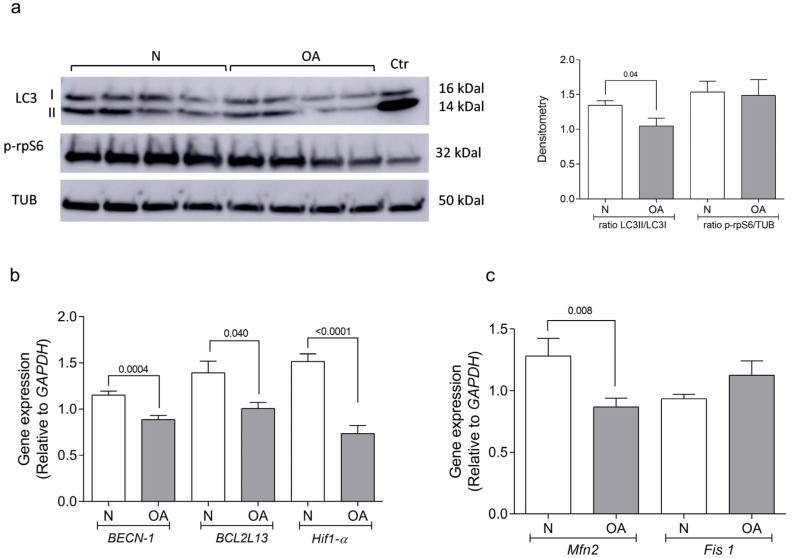
Autophagy Analysis. (**a**) Total protein from healthy (N) and osteoarthritic (OA) cybrids was examined using western blotting with antibodies against microtubule-associated protein 1A/1B-light chain 3 (LC3), phospho-ribosomal protein S6 (p-rpS6), and α-tubulin (TUB). Representative blots and densitometric quantification data are displayed. (**b**) Relative mRNA expression levels of beclin-1 (*BECN1*), B-cell lymphoma 2 homology motifs like protein 13 (*BCL2L13*), and hypoxia-inducible factor 1 subunit alpha (*Hif-1α*) were measured using quantitative real-time PCR (RT-PCR). (**c**) Relative mRNA expression levels of mitofusin 2 (*Mfn2*) and mitochondrial fission 1 (*Fis1*) were also assessed using RT-PCR. Data were collected from three independent experiments, each performed in duplicate, and are presented as mean ± SEM, analyzed by the unpaired Mann–Whitney U test. *GAPDH* (glyceraldehyde-3-phosphate dehydrogenase) was used as a reference. Positive controls were 30 µM chloroquine for LC3 and 10 µM rapamycin for p-rpS6.

Autophagy, a key homeostatic mechanism in cartilage, is reduced in OA chondrocytes [19,21]. To study this process in cybrids, LC3 protein was evaluated using the ratio LC3II/LC3I. Activation of the mTOR signaling pathway was measured for the analysis of p-rpS6, a downstream target of mTORC1 [17]. LC3 activation was found to be significantly reduced in OA compared to N cybrids (*p* = 0.04). However, the level of p-rpS6 did not differ between OA and N cybrids (Figure 3a). Full length uncropped original WB are represented in Supplementary Figure S1.

The levels of gene expression of beclin-1 (*BECN1*), an effector of autophagy, B-cell Lymphoma 2 homology motifs like protein 13 (*BCL2L13*), an autophagy promoter, and hypoxia inducible factor 1 subunit alpha (*Hif-1α*), a modulator of beclin-1/Bcl-2 complexes [39,40,41], were found to be significantly lower in OA cybrids compared to N cybrids (*p* = 0.0004 for *BECN1*, *p* = 0.04 for *BCL2L13*, and *p* < 0.0001 in the case of *Hif-1α*) (Figure 3b).

Alterations in autophagy flux were related to mitochondrial fragmentation and mitophagy, and were also necessary for mitochondrial fusion [42,43]. An analysis of the gene expression of mitofusin 2 (*Mfn2*) and mitochondrial fission-1 (*Fis 1*) under metabolic environmental stress (glucose, 1 g/L) revealed a lower genetic level in OA than N cybrids in the analysis of *Mfn2* (*p* = 0.008). However, levels of *Fis 1* did not show differences between OA and N cybrids (Figure 3c).

### 3.3. Senescence Analysis

**Figure 4:**
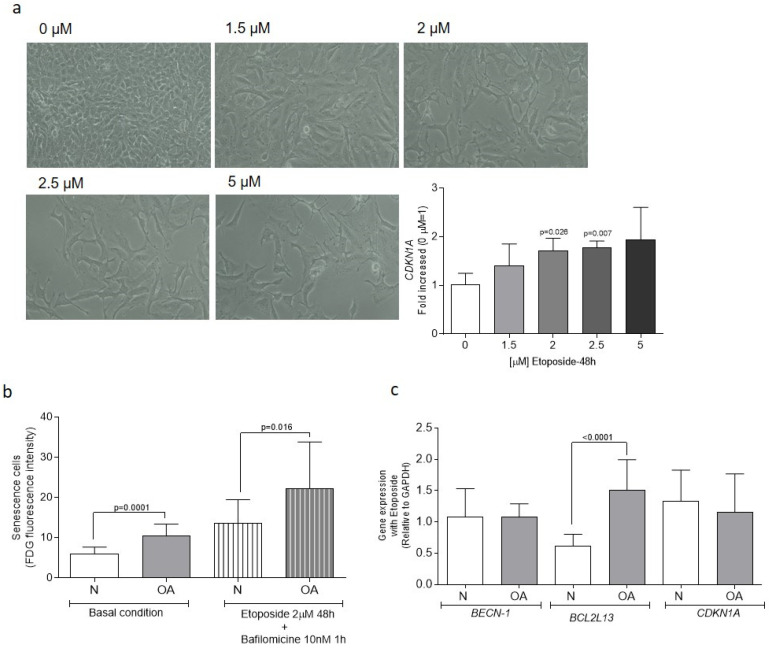
Analysis of Senescent Cells. (**a**) Determination of the optimal etoposide concentration was based on the gene expression of cyclin-dependent kinase inhibitor 1A (*CDKN1*) and the cellular morphology of cybrids treated with 0 and 2 µM etoposide over 48 h. (**b**) Senescent cells were assessed by β-galactosidase-mediated hydrolysis of fluorescein di-β-D-galactopyranoside (FDG), resulting in increased fluorescence measured by flow cytometry. (**c**) Relative mRNA expression levels of beclin-1 (*BECN1*), B-cell lymphoma 2 homology motifs like protein 13 (*BCL2L13*), and cyclin-dependent kinase inhibitor 1A (*CDKN1*) were measured using quantitative real-time PCR (RT-PCR). Data were collected from three independent experiments, each performed in duplicate, and are presented as mean ± SEM, analyzed by the unpaired Mann–Whitney U test. *GAPDH* served as a reference gene. N: healthy cybrids; OA: osteoarthritis cybrids.

Senescent cells were evaluated through the hydrolysis of FDG mediated by β-galactosidase and the gene expression of *CDKN1A*. Cultured cybrids were treated with either 0 to 5 μM etoposide for 48 h in order to determine the optimal concentration for increasing the percentage of senescent cells. It was found that 2 μM was enough to induce a phenotypic change and a significant increase in *CDKN1A* gene expression in comparison to untreated cells (*p* = 0.026) (Figure 4a). An analysis of basal conditions showed a significantly higher percentage of senescent cells in OA than in N cybrids (*p* = 0.0001) (Figure 4b). Following the increase in senescence described in OA cybrids, when cells were pretreated with etoposide in combination with bafilomycin A, higher levels of senescent cells were found in OA than N cybrids (*p* = 0.016) (Figure 4b).

Gene expression analysis of *BECN1*, *BCL2L13*, and *CDKN1A* showed that only levels of *BCL2L13* increased in OA cybrids in the presence of etoposide (*p* < 0.0001). However, levels of *BECN1* and *CDKN1A* were not modulated during the induction of senescence (Figure 4c).

## 4. Discussion

We found that mitochondria from OA donors are involved in three processes related to cartilage degradation in OA: cellular apoptosis, senescence, and autophagy.

Apoptosis has been positively correlated with the severity of cartilage damage and matrix depletion in human OA tissue [10,16,17]. In 2004, Roach and co-workers [44] coined the term “chondroptosis” to describe cells undergoing apoptosis in a non-classical manner that appeared to be typical of programmed chondrocyte death in vivo. We found that cybrids carrying mitochondria from OA patients were more susceptible to undergoing apoptosis than cybrids carrying mitochondria from individuals without OA when cultured in the presence of staurosporine and IL-1ẞ. Since OA cybrids behaved similarly to OA chondrocytes, this finding suggests a connection between OA mitochondria, the apoptotic pathway in OA, and chondrocyte/cartilage degradation [45].

Elevated reactive oxygen species (ROS) levels contribute to the dysregulation of tissue homeostasis and OA severity [46]. This heightened oxidative stress stems from mitochondrial dysfunction, as evidenced by OA chondrocytes displaying decreased mitochondrial DNA content and reduced mitochondrial mass [47]. To test survival capacity under mitochondrial stress, menadione was used. Menadione induced a pro-oxidative stress by producing intra-cellular H_2_O_2_, which induces oxidative stress, mitochondrial dysfunction, and apoptosis in endplate chondrocytes [48]. The presence of this compound increased the percentage of apoptotic cells in OA cybrids in comparison with N cybrids; when the activation of antioxidant response was evaluated, the gene expression of *SOD2* and *NFE2L2* decreased in OA cybrids. The redox-sensitive signaling system, Keap1/*NFE2L2*/ARE, plays a key role in the maintenance of cellular homeostasis under stress, and in inflammatory, carcinogenic, and proapoptotic conditions. *NFE2L2* plays a significant role in maintaining the structural and functional integrity of mitochondria, with its importance heightened especially during periods of stress [49]. The disturbance in mitochondrial function, leading to elevated levels of intracellular ROS, has been theorized to perturb cartilage homeostasis and contribute to the observed cartilage damage in OA [23,50]. Moreover, it has been reported that the mechanical loading of chondrocytes in vitro and in vivo promotes the mitochondrial generation of O^-^_2_· accompanied by a decrease in the expression of mitochondrial *SOD2* [7,51,52]. The aforementioned data highlighted that mitochondrial dysfunction in OA is associated with the downregulation of *SOD2*.

*Nrf1*, a transcription factor associated with *PPARGC1A*, plays a pivotal role in mitochondrial biogenesis. In the presence of menadione, cybrids exhibit differential expression of the *Nrf1* gene: N cybrids demonstrate elevated expression compared to OA cybrids. The observed expression pattern, coupled with prior research [37], indicates a potential deficit in mitochondrial biogenesis within OA cybrids. This aligns with observations in human OA chondrocytes, where impaired mitochondrial biogenesis is known to drive catabolic responses [47].

Apoptosis, characterized as programmed cell death, is acknowledged as a significant occurrence in the progression of OA. Meanwhile, autophagy also holds significance for cartilage homeostasis, playing a vital role in sustaining regular cellular metabolism, particularly in cell types like chondrocytes, which possess limited regenerative capabilities. Recent research indicates the potential co-occurrence of autophagy alongside apoptosis in OA [53]. Numerous studies have demonstrated the relationship between autophagy and the pathogenesis of OA, and a low level of autophagy was observed in senescent chondrocytes and is associated with OA cartilage [20,21,22,54]. Our data showing that OA cybrids have a lower LC3 activation and lower levels of beclin-1, Bcl-2-like protein 13, and hypoxia inducible factor-1α than N cybrids supports this idea. Low expression levels of *BECN1* may contribute toward chondrocyte death. *BECN1* overexpression in OA cartilage increased cell viability, inhibited matrix metalloproteinases, and inhibited chondrocyte apoptosis via PI3K/Akt/mTOR signaling [55]. Recently, it was found that *BCL2L13* binds to LC3 inducing mitochondrial fragmentation and mitophagy in other cell lines [42,43]. *Hif-1α* maintains homeostasis in chondrocytes [56] and mediates mitophagy, having a protective role in several diseases [57]. In chondrocytes, autophagy was stimulated by *Hif-1α* and the regulation of apoptosis by this gene was also described [39,40]. The mechanisms of autophagy induction by *Hif-1α* may involve the modulation of a beclin-1/Bcl-2 complex [57].

Mitochondria form a dynamic network characterized by tubular structures that undergo constant remodeling. *Mfn2*, a GTPase located in the outer mitochondrial membrane, plays a pivotal role in facilitating mitochondrial fusion. This process influences mitochondrial dynamics, distribution, quality control, and overall function [58]. Fusion serves various vital functions within cells, aiding in the alleviation of cellular stress by facilitating the removal of damaged mitochondria through a process known as mitophagy [58]. Our findings highlighted how OA cybrids show alterations in the autophagic process and in *Mfn2* gene expression. They are also likely to have an increase in damaged mitochondria, leading to an increase in metabolic disorder and inflammation as occurs during the OA process [59].

Senescent cells exhibit heightened levels of senescence-associated beta-galactosidase activity and comprise non-proliferative cell populations due to an irreversible state of growth arrest [60,61]. To promote and support cell-cycle arrest, p16INK4A (*CDKN2A*), accompanied by the p53 (*TP53*) target, p21 (*CDKN1A*), inhibits cyclin-dependent kinases, thereby preventing phosphorylation of the retinoblastoma protein and thus in turn suppressing the expression of proliferation-associated genes [62,63]. We found that OA cybrids have a higher number of senescent cells than N cybrids. Senescent cells exhibit metabolic activity, yet they accumulate dysfunctional mitochondria, leading to amplified mitochondrial mass, heightened oxygen consumption, diminished efficiency of oxidative phosphorylation, reduced membrane potential, and increased levels of ROS [26,28,64], as observed in OA cybrids [14,37]. These findings elucidate the role of mitochondrial alterations in inducing the senescence-associated secretory phenotype (SASP) during senescence [27,28]. OA is associated with a substantial presence of senescent cells, and SASP has been implicated in the degradation of cartilage [65,66].

Senescent cells also exhibit resistance to mitochondria-mediated apoptosis, in part by the upregulation of anti-apoptotic *BCL2* family members [54,55]. Similar findings were described, in this work, in OA cybrids where cells harboring mitochondria from OA donors in the presence of etoposide, a senescence inductor, showed an increase in the gene expression of *BCL2L13*.

The critical role of mitochondria in cellular senescence was described, and senescent cells accumulated dysfunctional mitochondria [27,64]. In OA, the presence of mitochondrial dysfunction was described [25,67,68], as was the rise in the number of senescent cells [30,65,69]. Taking into account the data described here, using an in vitro model with cells harboring OA mitochondria, the relevance of all these events in the OA process are related directly to OA mitochondria.

**Figure 5:**
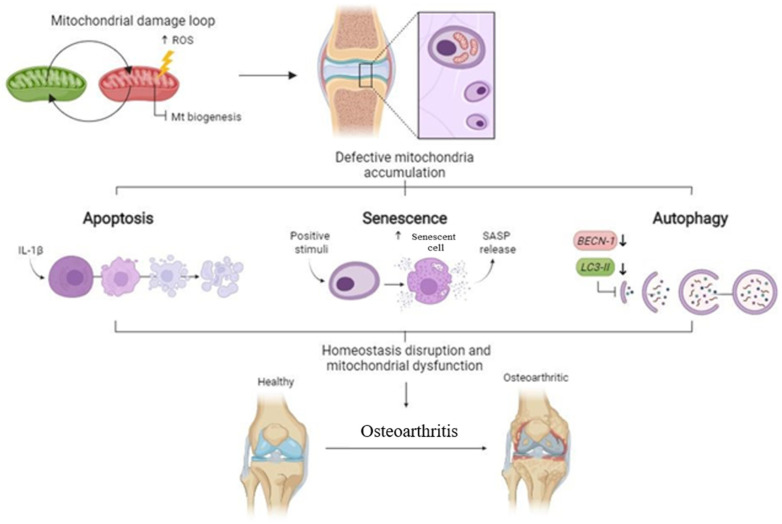
Scheme representing how the mitochondrial damage increases the mitochondria defective accumulation, and how this process is related with apoptosis, senescence, and autophagy, disrupting the mitochondrial function and the elimination of damage mitochondria. All these alterations break the equilibrium between anabolic and catabolic process in the cartilage and are related with the pathogenesis of OA.

Mitochondria have been described as playmakers of apoptosis, autophagy, and senescence [70], three processes that have also been detailed in OA. Using cybrids, this work suggests that mitochondria from OA patients are involved in the apoptosis, autophagy, and senescence described in OA cartilage (Figure 5).

The present work has some limitations. (1) This study could be replicated using a higher number of donors to analyze the reproducibility of the data. (2) The cybrids have a nuclear background derived from 143B.TK-cells. To evaluate these parameters and their relation to the osteoarthritis process, future research could generate cybrids with a chondrocyte nuclear background. (3) There might be differences between mitochondria from platelet and chondrocyte. If the mitochondrial dysfunction of the platelets was induced by OA processes, there could be a change in these parameters during permanent cell culture.

## Figures and Tables

**Table 1:** Gene name, sequence of primers, and UPL probes used in qRT-PCR amplification.

Gene Name	Symbol	Primer Fw (5′-3′)	Primer Rv (5′-3′)	UPL Probe
Nuclear factor erythroid 2 like 2	*NFE2L2*	gcaacaggacattgagcaag	tggacttggaaccatggtagt	#52
Superoxide dismutase-2	*SOD2*	ctggacaaacctcagcccta	tgatggcttccagcaactc	#22
Nuclear respiratory factor 1	*Nrf1*	ggggaaagaaagctgcaag	gtgcctgggtccatgaaa	#49
Beclin-1	*BECN-1*	caggctgaggctgagagact	gctccagctgctgtcgtt	#69
B-Cell Lymphoma 2 homology motifs like protein 13	*BCL2L13*	gacctcaacgcacagtacga	gagattgtacaggaccctcca	#68
Cyclin Dependent Kinase Inhibitor 1A	*CDKN1A*	tcactgtcttgtacccttgtg	ggcgtttggagtggtagaaa	#32
Hypoxia Inducible Factor 1 Subunit Alpha	*Hif-1α*	tggaatggagcaaaagacaa	tggtcagctgtggtaatcca	#3
Mitofusin 2	*Mfn-2*	tcagctacactggctccaac	caaaggtcccagacagttcc	#83
Mitochondrial Fusion 1	*Fis1*	ctgaacgagctggtgtctgt	gagcctgctgccttctca	#62
Glyceraldehyde-3-Phosphate Dehydrogenase	*GAPDH*	gagtccactggcgtcttcac	gttcacacccatgacgaaca	#45

Fw: Forward. Rv: Reverse. UPL Probe: Universal Probe Library probes.

## Data Availability

The original contributions presented in the study are included in the article/Supplementary Materials, further inquiries can be directed to the corresponding author/s.

## Supplementary Materials

The following supporting information can be downloaded at: https://www.mdpi.com/article/10.3390/cells13110976/s1, Figure S1: Full-length uncropped original WB.

## References

[1] Bannuru R.R., Osani M.C., Vaysbrot E.E., Arden N.K., Bennell K., Bierma-Zeinstra S.M.A., Kraus V.B., Lohmander L.S., Abbott J.H., Bhandari M. (2019). OARSI guidelines for the non-surgical management of knee, hip, and polyarticular osteoarthritis. Osteoarthr. Cartil..

[2] Kraus V.B., Blanco F.J., Englund M., Henrotin Y., Lohmander L.S., Losina E., Önnerfjord P., Persiani S. (2015). OARSI Clinical Trials Recommendations: Soluble biomarker assessments in clinical trials in osteoarthritis. Osteoarthr. Cartil..

[3] Loeser R.F., Goldring S.R., Scanzello C.R., Goldring M.B. (2012). Osteoarthritis: A disease of the joint as an organ. Arthritis Rheum..

[4] Blanco F.J. (2018). Osteoarthritis and atherosclerosis in joint disease. Reumatol. Clin..

[5] Haudenschild D.R., Carlson A.K., Zignego D.L., Yik J.H.N., Hilmer J.K., June R.K. (2019). Inhibition of early response genes prevents changes in global joint metabolomic profiles in mouse post-traumatic osteoarthritis. Osteoarthr. Cartil..

[6] Pelletier J.P., Martel-Pelletier J., Abramson S.B. (2001). Osteoarthritis, an inflammatory disease: Potential implication for the selection of new therapeutic targets. Arthritis Rheum..

[7] Bolduc J.A., Collins J.A., Loeser R.F. (2019). Reactive oxygen species, aging and articular cartilage homeostasis. Free Radic. Biol. Med..

[8] Zhu S., Makosa D., Miller B., Griffin T.M. (2020). Glutathione as a mediator of cartilage oxidative stress resistance and resilience during aging and osteoarthritis. Connect. Tissue Res..

[9] Collins J.A., Wood S.T., Nelson K.J., Rowe M.A., Carlson C.S., Chubinskaya S., Poole L.B., Furdui C.M., Loeser R.F. (2016). Oxidative Stress Promotes Peroxiredoxin Hyperoxidation and Attenuates Pro-survival Signaling in Aging Chondrocytes. J. Biol. Chem..

[10] Loeser R.F., Collins J.A., Diekman B.O. (2016). Ageing and the pathogenesis of osteoarthritis. Nat. Rev. Rheumatol..

[11] Zhao Z., Li Y., Wang M., Jin Y., Liao W., Zhao Z., Fang J. (2020). Mitochondrial DNA haplogroups participate in osteoarthritis: Current evidence based on a meta-analysis. Clin. Rheumatol..

[12] Fang H., Zhang F., Li F., Shi H., Ma L., Du M., You Y., Qiu R., Nie H., Shen L. (2016). Mitochondrial DNA haplogroups modify the risk of osteoarthritis by altering mitochondrial function and intracellular mitochondrial signals. Biochim. Biophys. Acta.

[13] Rego-Pérez I., Fernández-Moreno M., Fernández-López C., Arenas J., Blanco F.J. (2008). Mitochondrial DNA haplogroups: Role in the prevalence and severity of knee osteoarthritis. Arthritis Rheum..

[14] Fernández-Moreno M., Soto-Hermida A., Vázquez-Mosquera M.E., Cortés-Pereira E., Relaño S., Hermida-Gómez T., Pértega S., Oreiro-Villar N., Fernández-López C., Garesse R. (2017). Mitochondrial DNA haplogroups influence the risk of incident knee osteoarthritis in OAI and CHECK cohorts. A meta-analysis and functional study. Ann. Rheum. Dis..

[15] Cortés-Pereira E., Fernández-Tajes J., Fernández-Moreno M., Vázquez-Mosquera M.E., Relaño S., Ramos-Louro P., Durán-Sotuela A., Dalmao-Fernández A., Oreiro N., Blanco F.J. (2019). Differential Association of Mitochondrial DNA Haplogroups J and H with the Methylation Status of Articular Cartilage: Potential Role in Apoptosis and Metabolic and Developmental Processes. Arthritis Rheumatol..

[16] Blanco F.J., Guitian R., Vázquez-Martul E., de Toro F.J., Galdo F. (1998). Osteoarthritis chondrocytes die by apoptosis. A possible pathway for osteoarthritis pathology. Arthritis Rheum..

[17] Musumeci G., Castrogiovanni P., Trovato F.M., Weinberg A.M., Al-Wasiyah M.K., Alqahtani M.H., Mobasheri A. (2015). Biomarkers of Chondrocyte Apoptosis and Autophagy in Osteoarthritis. Int. J. Mol. Sci..

[18] Carroll B., Nelson G., Rabanal-Ruiz Y., Kucheryavenko O., Dunhill-Turner N.A., Chesterman C.C., Zahari Q., Zhang T., Conduit S.E., Mitchell C.A. (2017). Persistent mTORC1 signaling in cell senescence results from defects in amino acid and growth factor sensing. J. Cell Biol..

[19] Li Y.S., Zhang F.J., Zeng C., Luo W., Xiao W.F., Gao S.G., Lei G.H. (2016). Autophagy in osteoarthritis. Jt. Bone Spine.

[20] Lotz M.K., Caramés B. (2011). Autophagy and cartilage homeostasis mechanisms in joint health, aging and OA. Nat. Rev. Rheumatol..

[21] Caramés B., Olmer M., Kiosses W.B., Lotz M.K. (2015). The relationship of autophagy defects to cartilage damage during joint aging in a mouse model. Arthritis Rheumatol..

[22] Caramés B., Taniguchi N., Otsuki S., Blanco F.J., Lotz M. (2010). Autophagy is a protective mechanism in normal cartilage, and its aging-related loss is linked with cell death and osteoarthritis. Arthritis Rheumatol..

[23] Blanco F.J., Rego I., Ruiz-Romero C. (2011). The role of mitochondria in osteoarthritis. Nat. Rev. Rheumatol..

[24] Kan S., Duan M., Liu Y., Wang C., Xie J. (2021). Role of Mitochondria in Physiology of Chondrocytes and Diseases of Osteoarthritis and Rheumatoid Arthritis. Cartilage.

[25] Blanco F.J., Rego-Pérez I. (2018). Mitochondria and mitophagy: Biosensors for cartilage degradation and osteoarthritis. Osteoarthr. Cartil..

[26] Passos J.F., Nelson G., Wang C., Richter T., Simillion C., Proctor C.J., Miwa S., Olijslagers S., Hallinan J., Wipat A. (2010). Feedback between p21 and reactive oxygen production is necessary for cell senescence. Mol. Syst. Biol..

[27] Correia-Melo C., Marques F.D., Anderson R., Hewitt G., Hewitt R., Cole J., Carroll B.M., Miwa S., Birch J., Merz A. (2016). Mitochondria are required for pro-ageing features of the senescent phenotype. EMBO J..

[28] Vizioli M.G., Liu T., Miller K.N., Robertson N.A., Gilroy K., Lagnado A.B., Perez-Garcia A., Kiourtis C., Dasgupta N., Lei X. (2020). Mitochondria-to-nucleus retrograde signaling drives formation of cytoplasmic chromatin and inflammation in senescence. Genes Dev..

[29] Mobasheri A., Matta C., Zákány R., Musumeci G. (2015). Chondrosenescence: Definition, hallmarks and potential role in the pathogenesis of osteoarthritis. Maturitas.

[30] Martin J.A., Brown T.D., Heiner A.D., Buckwalter J.A. (2004). Chondrocyte senescence, joint loading and osteoarthritis. Clin. Orthop. Relat. Res..

[31] Youle R.J., van der Bliek A.M. (2012). Mitochondrial fission, fusion, and stress. Science.

[32] Liesa M., Palacín M., Zorzano A. (2009). Mitochondrial dynamics in mammalian health and disease. Physiol. Rev..

[33] Zorzano A., Liesa M., Sebastián D., Segalés J., Palacín M. (2010). Mitochondrial fusion proteins: Dual regulators of morphology and metabolism. Semin. Cell Dev. Biol..

[34] Chan D.C. (2012). Fusion and fission: Interlinked processes critical for mitochondrial health. Annu. Rev. Genet..

[35] Vanden Berghe T., Kaiser W.J., Bertrand M.J., Vandenabeele P. (2015). Molecular crosstalk between apoptosis, necroptosis, and survival signaling. Mol. Cell. Oncol..

[36] Dalmao-Fernández A., Lund J., Hermida-Gómez T., Vazquez-Mosquera M.E., Rego-Pérez I., Blanco F.J., Fernández-Moreno M. (2020). Impaired Metabolic Flexibility in the Osteoarthritis Process: A Study on Transmitochondrial Cybrids. Cells.

[37] Dalmao-Fernández A., Hermida-Gómez T., Lund J., Vazquez-Mosquera M.E., Rego-Pérez I., Garesse R., Blanco F.J., Fernández-Moreno M. (2021). Mitochondrial DNA from osteoarthritic patients drives functional impairment of mitochondrial activity: A study on transmitochondrial cybrids. Cytotherapy.

[38] Loor G., Kondapalli J., Schriewer J.M., Chandel N.S., Vanden Hoek T.L., Schumacker P.T. (2010). Menadione triggers cell death through ROS-dependent mechanisms involving PARP activation without requiring apoptosis. Free Radic. Biol. Med..

[39] Bohensky J., Shapiro I.M., Leshinsky S., Terkhorn S.P., Adams C.S., Srinivas V. (2007). HIF-1 regulation of chondrocyte apoptosis: Induction of the autophagic pathway. Autophagy.

[40] Bohensky J., Leshinsky S., Srinivas V., Shapiro I.M. (2010). Chondrocyte autophagy is stimulated by HIF-1 dependent AMPK activation and mTOR suppression. Pediatr. Nephrol..

[41] Zhang F., Wang J., Chu J., Yang C., Xiao H., Zhao C., Sun Z., Gao X., Chen G., Han Z. (2015). MicroRNA-146a Induced by Hypoxia Promotes Chondrocyte Autophagy through Bcl-2. Cell. Physiol. Biochem. Int. J. Exp. Cell. Physiol. Biochem. Pharmacol..

[42] Otsu K., Murakawa T., Yamaguchi O. (2015). BCL2L13 is a mammalian homolog of the yeast mitophagy receptor Atg32. Autophagy.

[43] Murakawa T., Yamaguchi O., Hashimoto A., Hikoso S., Takeda T., Oka T., Yasui H., Ueda H., Akazawa Y., Nakayama H. (2015). Bcl-2-like protein 13 is a mammalian Atg32 homologue that mediates mitophagy and mitochondrial fragmentation. Nat. Commun..

[44] Roach H.I., Aigner T., Kouri J.B. (2004). Chondroptosis: A variant of apoptotic cell death in chondrocytes?. Apoptosis Int. J. Program. Cell Death.

[45] Kim H.A., Lee Y.J., Seong S.C., Choe K.W., Song Y.W. (2000). Apoptotic chondrocyte death in human osteoarthritis. J. Rheumatol..

[46] Hui W., Young D.A., Rowan A.D., Xu X., Cawston T.E., Proctor C.J. (2016). Oxidative changes and signalling pathways are pivotal in initiating age-related changes in articular cartilage. Ann. Rheum. Dis..

[47] Wang Y., Zhao X., Lotz M., Terkeltaub R., Liu-Bryan R. (2015). Mitochondrial biogenesis is impaired in osteoarthritis chondrocytes but reversible via peroxisome proliferator-activated receptor γ coactivator 1α. Arthritis Rheumatol..

[48] Kang L., Liu S., Li J., Tian Y., Xue Y., Liu X. (2020). Parkin and Nrf2 prevent oxidative stress-induced apoptosis in intervertebral endplate chondrocytes via inducing mitophagy and anti-oxidant defenses. Life Sci..

[49] Dinkova-Kostova A.T., Abramov A.Y. (2015). The emerging role of Nrf2 in mitochondrial function. Free Radic. Biol. Med..

[50] Terkeltaub R., Johnson K., Murphy A., Ghosh S. (2002). Invited review: The mitochondrion in osteoarthritis. Mitochondrion.

[51] Ruiz-Romero C., Calamia V., Mateos J., Carreira V., Martínez-Gomariz M., Fernández M., Blanco F.J. (2009). Mitochondrial dysregulation of osteoarthritic human articular chondrocytes analyzed by proteomics: A decrease in mitochondrial superoxide dismutase points to a redox imbalance. Mol. Cell. Proteom. MCP.

[52] Gavriilidis C., Miwa S., von Zglinicki T., Taylor R.W., Young D.A. (2013). Mitochondrial dysfunction in osteoarthritis is associated with down-regulation of superoxide dismutase 2. Arthritis Rheumatol..

[53] Almonte-Becerril M., Navarro-Garcia F., Gonzalez-Robles A., Vega-Lopez M.A., Lavalle C., Kouri J.B. (2010). Cell death of chondrocytes is a combination between apoptosis and autophagy during the pathogenesis of Osteoarthritis within an experimental model. Apoptosis Int. J. Program. Cell Death.

[54] Vinatier C., Domínguez E., Guicheux J., Caramés B. (2018). Role of the Inflammation-Autophagy-Senescence Integrative Network in Osteoarthritis. Front. Physiol..

[55] Ryu S.J., Oh Y.S., Park S.C. (2007). Failure of stress-induced downregulation of Bcl-2 contributes to apoptosis resistance in senescent human diploid fibroblasts. Cell Death Differ..

[56] Schipani E., Ryan H.E., Didrickson S., Kobayashi T., Knight M., Johnson R.S. (2001). Hypoxia in cartilage: HIF-1alpha is essential for chondrocyte growth arrest and survival. Genes Dev..

[57] Hu S., Zhang C., Ni L., Huang C., Chen D., Shi K., Jin H., Zhang K., Li Y., Xie L. (2020). Stabilization of HIF-1α alleviates osteoarthritis via enhancing mitophagy. Cell Death Dis..

[58] Filadi R., Pendin D., Pizzo P. (2018). Mitofusin 2: From functions to disease. Cell Death Dis..

[59] Blanco F.J., Fernández-Moreno M. (2020). Mitochondrial biogenesis: A potential therapeutic target for osteoarthritis. Osteoarthr. Cartil..

[60] Collado M., Blasco M.A., Serrano M. (2007). Cellular senescence in cancer and aging. Cell.

[61] Campisi J., d’Adda di Fagagna F. (2007). Cellular senescence: When bad things happen to good cells. Nat. Rev. Mol. Cell Biol..

[62] Yosef R., Pilpel N., Papismadov N., Gal H., Ovadya Y., Vadai E., Miller S., Porat Z., Ben-Dor S., Krizhanovsky V. (2017). p21 maintains senescent cell viability under persistent DNA damage response by restraining JNK and caspase signaling. EMBO J..

[63] Ock S.A., Knott J.G., Choi I. (2020). Involvement of CDKN1A (p21) in cellular senescence in response to heat and irradiation stress during preimplantation development. Cell Stress Chaperones.

[64] Korolchuk V.I., Miwa S., Carroll B., von Zglinicki T. (2017). Mitochondria in Cell Senescence: Is Mitophagy the Weakest Link?. EBioMedicine.

[65] Coryell P.R., Diekman B.O., Loeser R.F. (2021). Mechanisms and therapeutic implications of cellular senescence in osteoarthritis. Nat. Rev. Rheumatol..

[66] Astrike-Davis E.M., Coryell P., Loeser R.F. (2022). Targeting cellular senescence as a novel treatment for osteoarthritis. Curr. Opin. Pharmacol..

[67] Rego-Pérez I., Durán-Sotuela A., Ramos-Louro P., Blanco F.J. (2019). Mitochondrial Genetics and Epigenetics in Osteoarthritis. Front. Genet..

[68] Zheng L., Zhang Z., Sheng P., Mobasheri A. (2021). The role of metabolism in chondrocyte dysfunction and the progression of osteoarthritis. Ageing Res. Rev..

[69] Loeser R.F. (2009). Aging and osteoarthritis: The role of chondrocyte senescence and aging changes in the cartilage matrix. Osteoarthr. Cartil..

[70] Abate M., Festa A., Falco M., Lombardi A., Luce A., Grimaldi A., Zappavigna S., Sperlongano P., Irace C., Caraglia M. (2020). Mitochondria as playmakers of apoptosis, autophagy and senescence. Semin. Cell Dev. Biol..

