# Supplementary figures and images for: Mitochondrial Role on Cellular Apoptosis, Autophagy, and Senescence during Osteoarthritis Pathogenesis
Source: Cells. 2024 Jun 4;13(11):976. doi: 10.3390/cells13110976 (PMC11172191; doi:10.3390/cells13110976)

LC3

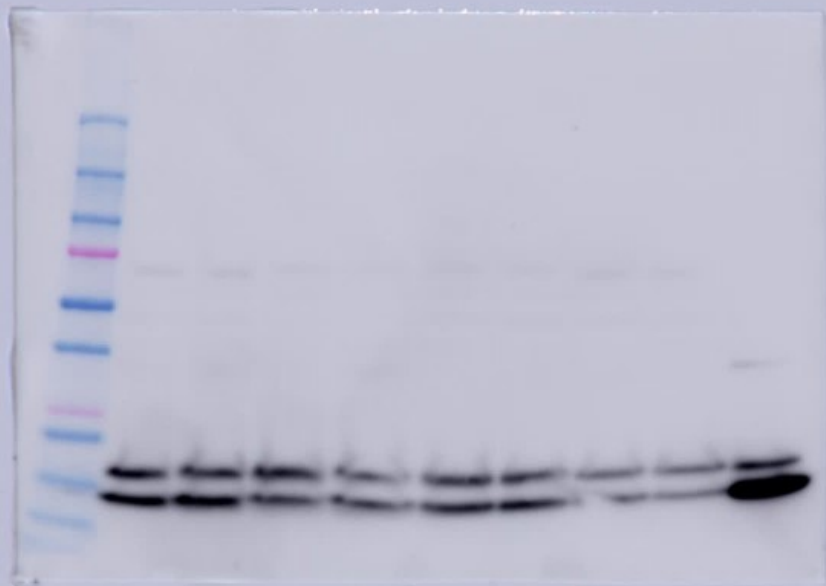

p-rpS6

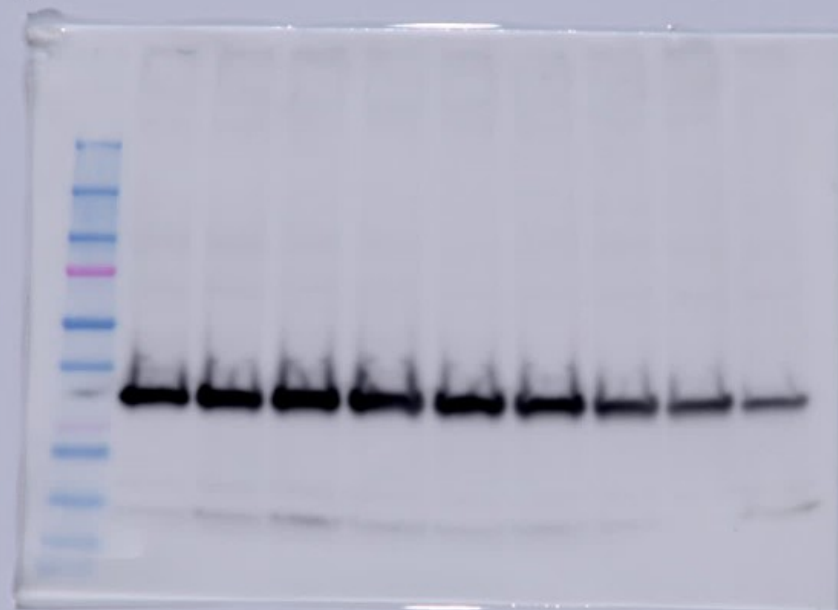

$\alpha$ -Tubulin (TUB)

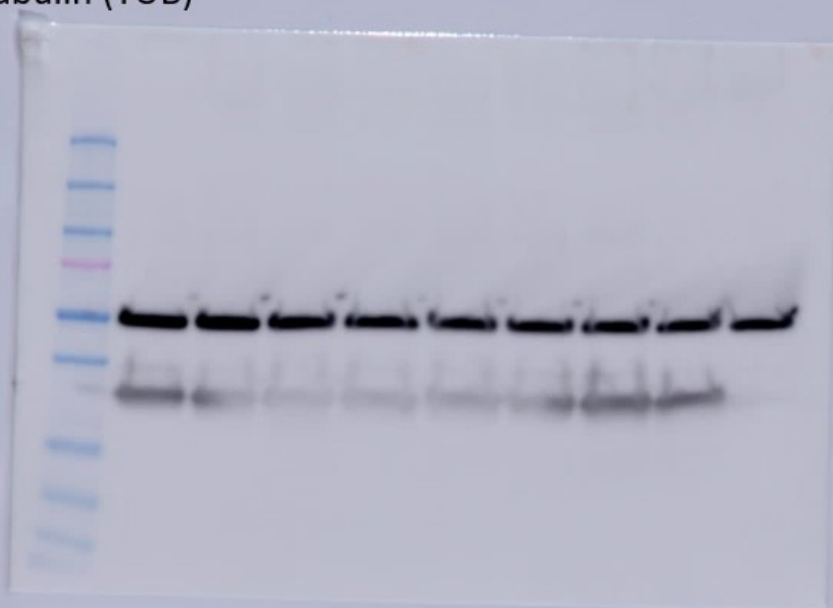

Supplement: Supplementary file 1 [file cells-13-00976-s001.zip › cells-2972192-supplementary.pdf]
